# Asthma

**DOI:** 10.1186/s13223-025-00949-4

**Published:** 2025-02-10

**Authors:** Andrew O’Keefe, Lori Connors, Ling Ling, Harold Kim

**Affiliations:** 1 Memorial University, St. John’s, NF Canada; 2 Department of Medicine, Dalhousie University, Halifax, NS Canada; 3 Department of Medicine, University of Alberta, Edmonton, AB Canada; 4 Division of Allergy and Immunology, Department of Medicine, Western University, London, ON Canada; 5 Division of Allergy and Immunology, Department of Medicine, McMaster University, Hamilton, ON Canada

## Abstract

Asthma is one of the most common respiratory disorders in Canada, however, many Canadians with asthma remain poorly controlled. In most patients, control can be achieved through appropriate therapy, including: inhaled corticosteroids (ICS), combination ICS/long-acting beta_2_-agonists (LABA), “triple therapy” with ICS/LABA/long-acting muscarinic receptor antagonist (LAMA), and biologic therapies. The medical management of severe asthma, in particular, has changed dramatically with the incorporation of biologics in asthma treatment plans. Allergen-specific immunotherapy represents a potentially disease-modifying therapy for many patients with asthma; it must only be prescribed by physicians with appropriate training in allergy. Other essential components of asthma management include: regular monitoring of asthma control and risk of exacerbations; patient education and written asthma action plans; assessing barriers to treatment and adherence to therapy; adequate management of comorbidities (e.g., allergic rhinitis) and reviewing inhaler device technique. This article provides a review of current literature and guidelines for the appropriate diagnosis and management of asthma in adults and children.

## Key take-home messages

- A clinical diagnosis of asthma should be suspected in patients with intermittent symptoms of wheezing, coughing, chest tightness and breathlessness.
- Objective measurements of lung function, preferably using spirometry, are needed to confirm the diagnosis. The best time to perform this testing is when the patient is symptomatic. Spirometry can generally be performed in children 6 years of age and older.
- In children < 6 years of age who are unable to perform spirometry, a trial of therapy (8-12 weeks in duration) and monitoring of symptoms can act as a surrogate method to diagnose asthma.
- All asthma patients should be prescribed a rapid-acting bronchodilator to be used as needed for relief of acute symptoms.
- ICS therapy (either alone or in combination with a LABA) is the standard of care for most patients with asthma.
- LTRAs can also be used as add-on therapy if asthma is uncontrolled despite the use of low-to-moderate ICS or ICS/LABA doses.
- The LAMA, tiotropium, by mist inhaler can be added in patients 18 years of age or older with an exacerbation history despite ICS/LABA treatment. Two “triple therapy” combination ICS/LABA/LAMA inhalers are approved for use in Canadian adults.
- Biologic therapy targeting IgE, IL-4/IL-13, IL-5 or TSLP may be useful in select cases of difficult to control or severe asthma.
- Allergen-specific immunotherapy is a potentially disease-modifying therapy that can be considered in most cases of allergic asthma.
- Regular monitoring of asthma control and exacerbation risk, adherence to therapy and inhaler technique are important components of asthma management.

## Introduction

Asthma affects approximately 11% of the Canadian population. It is one of the most common chronic respiratory diseases and the most common chronic disease of childhood [1]. Although asthma is often believed to be a disorder localized to the lungs, it represents a component of systemic airway disease, and frequently coexists with other atopic disorders, particularly allergic rhinitis (see *Allergic Rhinitis* article in this supplement) [2], atopic dermatitis (see *Atopic Dermatitis* article in this supplement) [3] and chronic rhinosinusitis with nasal polyposis (CRSwNP) [4].

Despite significant improvements in the diagnosis and management of asthma, asthma control in Canada remains suboptimal. Results from the Reality of Asthma Control in Canada study suggest that over 50% of Canadians with asthma have uncontrolled disease [5]. In another Canadian study that included 503 patients with severe asthma, approximately 47% reported ≥ 2 exacerbations requiring oral corticosteroids (OCS) and 14% required hospitalization due to an exacerbation in the past 12 months [6]. The longitudinal Canadian SABA in Asthma (SABINA) study that assessed over 115,400 asthma patients found that 39% of patients in Nova Scotia and 28% in Alberta overuse SABA therapy (defined as ≥ 3 canisters per year), which was associated an increased risk of severe exacerbations [7]. SABA overuse is a key marker of poor asthma control, and poor control contributes to unnecessary morbidity, limitations to daily activities and impairments in overall quality of life [8].

This article provides an overview of recent diagnostic and therapeutic guideline recommendations from the Global Initiative for Asthma (GINA) [9] and the Canadian Thoracic Society (CTS) [10] as well as current literature related to the pathophysiology, diagnosis, and appropriate treatment of asthma.

### Definition

According to GINA, asthma is a heterogeneous disease, usually characterized by chronic airway inflammation [9]. It is defined by a history of respiratory symptoms, such as wheeze, shortness of breath, chest tightness and cough, that vary over time and in intensity, together with variable expiratory airflow limitation [9].

### Epidemiology

Epidemiological evidence suggests that the prevalence of asthma in Canada is rising. In Alberta, the age-adjusted prevalence of asthma increased threefold between 1995 and 2015 (for both genders), from 3.9 to 12.3% in females and from 3.5 to 11.6% in males [11]. In British Columbia, the prevalence of asthma increased from 8% in 2001/02 to 12% in 2021/22 [12]. Similarly, data from the Ontario Asthma Surveillance Information System (OASIS) — a population-based longitudinal surveillance system which identifies and tracks individuals living with asthma — found an increase in asthma prevalence in Ontario, from 8.8 per 100 population in 1996 to 15.6 per 100 population in 2022 [13]. The results of these studies suggest that effective clinical and public health strategies are needed to prevent and manage asthma in the Canadian population.

### Pathophysiology

Given that asthma is a heterogenous disease, its pathophysiology is complex. Non-type 2 (non-T2) and T2 asthma have been described, with the latter also labelled as T2 high and T2 low asthma [14]. Some patients may begin with one asthma endotype and progress to another over time. The majority of patients with severe asthma are believed to have T2 high disease [14, 15]. T2 high asthma is associated with T helper cell type-2 (Th2) immune responses, which are typical of other atopic conditions. Triggers may include allergens (e.g., house dust mites, cockroach residue, animal dander, mould, and pollens), irritants, pollutants and infectious stimuli (e.g., viral infections, exposure to tobacco smoke, cold air, exercise), which produce a cascade of events leading to chronic airway inflammation. Elevated levels of Th2 cells in the airways release specific cytokines, including interleukin (IL)-4, IL-5, IL-9 and IL-13, and promote eosinophilic inflammation and immunoglobulin E (IgE) production. IgE production, in turn, triggers the release of inflammatory mediators, such as histamine and cysteinyl leukotrienes, that cause bronchospasm (contraction of the smooth muscle in the airways), edema, and increased mucous secretion, which lead to the characteristic symptoms of asthma [16]. Airway epithelium also produces alarmins and cytokines in response to infection, injury or irritant exposure. These cytokines include thymic stromal lymphopoietin (TSLP), IL-25 and IL-33, which activate type 2 innate lymphoid cells (IL-C2) [14]. IL-C2 then activates the T2 pathway discussed above.

T2 low asthma pathophysiology is not as well defined as T2 high asthma. IL-17 is thought to play an important role, which is secreted by CD4 + Th17 cells, as well as CD8 + T cells, NK cells, mucosal-associated invariant T (MAIT) cells and B cells [14]. IL-17 can stimulate fibroblasts and epithelial cells to release neutrophil chemoattractants, thus leading to neutrophilic inflammation in the airways. Some subtypes of IL-17 enhance airway smooth muscle contraction and proliferation, which in turn contributes to airway hyperresponsiveness and remodelling [14].

Our knowledge of the pathophysiology of asthma continues to evolve, and the role of other cytokines, protein kinases and other molecules in the pathophysiology of the disease are currently being studied [14].

### Diagnosis

An asthma diagnosis is based on *both* a clinical history and physical examination compatible with asthma *and* objective evidence of reversible airflow obstruction [9, 10]. Criteria for the diagnosis of asthma based on patient age is provided in Table 1 [10].

**Table 1:** CTS criteria for the diagnosis of asthma according to age [10]

	Clinical history compatible with asthma: paroxysmal or persistent symptoms such as dyspnea, chest tightness, wheezing, sputum production, and cough; AND confirmation of reversible airflow obstruction		
	Children (1–5 years of age)	Children (≥ 6 years of age)	Adults (≥ 18 years of age)
**Preferred**	Documentation by trained health care provider of wheeze and other signs of airflow obstruction with documented improvement with SABA ± oral corticosteroids	Spirometry showing reversible airflow obstruction FEV_1_/FVC < LLN (< 0.8–0.9^a^) AND increase in FEV_1_ after a bronchodilator or after a course of controller therapy of ≥ 12%	Spirometry showing reversible airflow obstruction FEV_1_/FVC < LLN (< 0.75–0.8^a^) AND increase in FEV_1_ after a bronchodilator or after a course of controller therapy of ≥ 12% and a minimum of ≥ 200 mL
**Alternative**	Convincing caregiver report of wheezing or other symptoms of airflow obstruction with symptomatic response to a 3-month trial of a medium dose of ICS and as needed SABA or symptomatic response to SABA^b^	PEF ≥ 20% increase after a bronchodilator or after a course of controller therapy^c^	PEF 60 L/min (minimum ≥ 20%) increase after a bronchodilator or after a course of controller therapy^c^ OR Diurnal variation > 8% based on twice daily readings; > 20% based on multiple daily readings^d^
**Alternative**		Positive challenge test Methacholine PC_20_ < 4 mg/ml or PD_20_ < 0.5 µmol (100 mcg) PC_20_ 4–16 mg/mL or PD_20_ 0.2–2 µmol (100–400 mcg) is borderline PC_20_ > 16 mg/mL or PD_20_ > 2 µmol (> 400 mcg) is negative OR Exercise challenge with ≥ 10–15% decrease in FEV_1_ post-exercise	

^a^ Approximate lower limits of normal ratios for children and adults

^b^ In children with mild intermittent symptoms and mild exacerbations, the diagnosis is only suggested because the accuracy of parental report of response to treatment may be unreliable due to misperception and spontaneous improvement of symptoms, which is why confirmation of reversible obstruction by direct observation from a health care provider is preferred

^c^ Comparison of PEF values should be done on the same device given the variability between devices

^d^ Difference between minimum AM pre-bronchodilator value in 1 week and maximum PM value as % of recent maximum

*SABA* short-acting beta-agonist, *FEV*_*1*_ forced expiratory volume in 1 s, *FVC* forced vital capacity, *LLN* lower limit of normal, *ICS* inhaled corticosteroid, *PC*_20_ provocative concentration, *PD*_20_ provocative dose, *PEF* peak expiratory flow

#### Medical history

Asthma should be suspected in patients with classic symptoms (wheeze, shortness of breath, chest tightness and/or cough) that are variable, occur upon exposure to triggers such as allergens or irritants, worsen at night and respond to asthma therapy [9, 10]. A family history of asthma or other atopic diseases and/or a personal history of atopic disorders can also be helpful in identifying patients with asthma.

During the history, it is also important to enquire about triggers of asthma symptoms, such as environmental allergens, exercise, and exposure to tobacco smoke or cold air. If occupational asthma is suspected, details of work exposures and improvements in asthma symptoms during time away from the workplace should be explored. Key questions to ask when taking the medical history of a patient with suspected asthma are provided in Table 2.

**Table 2:** Key questions to ask when taking the medical history of patients with suspected asthma

∙ Asthma symptoms (cough, wheeze, increased work of breathing)?
∙ Age of onset of symptoms?
∙ Timing of symptoms (day vs. night)?
∙ Is there a seasonal component to the worsening of symptoms?
∙ Possible triggers (viral infections, animal exposures, pollens, tobacco smoke, emotion)?
∙ Severity of symptoms (often reflected by unscheduled physician appointments at a walk-in clinic or emergency room, hospital admissions, and need for rescue oral corticosteroids)?
∙ Past investigations including chest x-rays, spirometry, allergy testing, sweat chloride testing?
∙ Other co-morbidities (e.g., allergic rhinitis, CRSwNP, food allergy, eosinophilic esophagitis, atopic dermatitis)?
∙ Current and past treatments? Duration of use? Reasons for discontinuation?
∙ Barriers to treatment (cost of medication, proximity to health care providers)?
∙ Exposure to second- and third-hand (i.e., the lingering smell of tobacco smoke on clothing or in vehicles) tobacco smoke?
∙ Presence of household pets?
∙ Impact of the symptoms on the patient/family quality of life (missed time from activities, school or work due to asthma symptoms)?

#### Physical examination

Physical examination in people with asthma is often normal [9]. Given the variability of asthma symptoms, physical findings may only be evident if the patient is symptomatic, and their absence does not exclude a diagnosis of asthma. The most common abnormal physical finding is expiratory wheezing on auscultation, but this may be absent or only heard on forced expiration [9].

#### Objective measurements to confirm variable expiratory airflow limitation

Objective evidence of excessive variability in expiratory airflow limitation is essential to confirming the diagnosis of asthma (see Table 1) [9, 10]. The greater the variations in lung function, or the more times excess variation is seen, the more likely the diagnosis is to be asthma.

In patients 6 years of age and over, lung function is most reliably assessed by spirometry, with assessment of forced expiratory volume in 1 second (FEV1) and the ratio of FEV1 to forced vital capacity (FEV1/FVC) pre and post administration of a bronchodilator [9, 10]. Patients will not exhibit reversible airway obstruction at every visit, and a negative spirometry result does not rule out a diagnosis of asthma [10]. This is particularly true for children who experience symptoms predominantly with viral infections. Repeating spirometry when patients are symptomatic can improve sensitivity. Even on repeat testing, spirometry may still be normal in some patients with asthma, so bronchoprovocation challenge testing and assessing for markers of airway inflammation may also be necessary.

#### Tests of bronchial hyperresponsiveness

When spirometry is normal, but symptoms and the clinical history are suggestive of asthma, measurement of airway responsiveness using direct airway challenges to inhaled bronchoconstrictor stimuli (e.g., methacholine or histamine) or indirect challenges (e.g., with mannitol or exercise) may help confirm a diagnosis of asthma [9, 10]. A Canadian study found that of 410 patients ultimately diagnosed with asthma, only 86 (20.9%) were diagnosed based on spirometry at the initial assessment while 287 (70%) were diagnosed based on bronchoprovocation testing [17].

Bronchoprovocation testing involves the patient inhaling increasing doses or concentrations of an inert stimulus until a given level of bronchoconstriction is achieved, typically a 20% fall in FEV_1_. An inhaled rapid-acting bronchodilator is then provided to reverse the obstruction. Positive challenge tests are not specific to asthma and may occur with other conditions such as allergic rhinitis and chronic obstructive pulmonary disease (COPD). Therefore, tests of bronchial hyperreactivity may be most useful for ruling out asthma among individuals who are symptomatic. A negative test result in a symptomatic patient not receiving anti-inflammatory therapy is highly sensitive [18].

#### Non-invasive markers of airway inflammation

The measurement of inflammatory markers such as sputum eosinophilia (proportion of eosinophils in the cell analysis of sputum) or fractional exhaled nitric oxide (FeNO) (nitric oxide is a gaseous molecule produced by some cells during an inflammatory response) can also be useful for diagnosing asthma. However, these tests are not yet widely available in Canada.

#### Allergy skin testing

For patients with suspected allergic asthma, skin prick (epicutaneous) testing to environmental allergens may be recommended to identify possible triggers [9]. There is no minimum age at which skin prick testing can be performed. Although allergen-specific IgE tests (i.e., measurement of the level of specific IgE in serum) have been suggested as an alternative to skin prick tests, these tests are more expensive and are no more reliable than skin prick tests [9]. However, they may be preferred for uncooperative patients or those with widespread skin disease [9]. It is important to note that the presence of a positive skin test or positive allergen-specific IgE does not mean that the allergen is causing symptoms – the relevance of allergen exposure and its relation to symptoms must be confirmed by the patient’s history [9].

#### Differential diagnosis

The differential diagnosis in a patient with suspected asthma varies with age (Table 3) [9, 19]. Conditions that should be considered in the differential diagnosis of adults with suspected asthma may include: COPD, bronchitis, gastrointestinal reflux disease, recurrent respiratory infections, heart disease, and vocal cord dysfunction (Table 3). It is important to note that any of these alternative diagnoses may also be found together with asthma. For example, a population-based cohort study conducted in Ontario suggests that the prevalence of concurrent asthma and COPD is increasing, particularly in women and young adults [20].

**Table 3:** Alternative diagnoses to consider in pediatric and adult patients with suspected asthma [9, 19]

Infections	Mechanical conditions	Congenital conditions
**Pediatric alternative diagnoses**		
· Recurrent repiratory tract infections · Persistent bacterial bronchitis · Chronic rhinosinusitis · Tuberculosis	· Foreign body aspiration · Gastroesophageal reflux	· Cystic fibrosis · Immune deficiency · Congenital heart disease · Bronchopulmonary dysplasia · Primary ciliary dyskinesia syndrome · Tracheomalacia
**Adult alternative diagnoses**		
· COPD · Infectious etiologies (bacterial, viral, fungal) · GERD · Chronic rhinosinusitis with or without nasal polyposis · CHF · Inducible laryngeal obstruction and other disorders of the upper airways	· Idiopathic anaphylaxis with predominant respiratory manifestations · Aspirin or nonsteroidal anti-inflammatory drug- exacerbated respiratory disease (AERD/NERD) · Sarcoidosis and other autoimmune processes · Pulmonary hypertension · Drug-induced bronchospasm	· Lymphangioleiomyomatosis (LAM) · Cystic fibrosis · Simple pulmonary eosinophilia (Loeffler’s syndrome) and other eosinophilic lung diseases · Vasculitides · Eosinophilic granulomatosis with polyangiitis · Granulomatosis with polyangiitis · Microscopic polyangiitis

*AERD* aspirin-exacerbated respiratory disease, *COPD* chronic obstructive pulmonary disease, *GERD* gastroesophageal reflux disease, *CHF* congestive heart failure, *NERD* nonsteroidal anti-inflammatory drug- exacerbated respiratory disease

The differential diagnosis of asthma is unique for infants and young children and includes infections (e.g., recurrent respiratory tract infections, chronic rhinosinusitis), mechanical conditions (e.g., foreign body aspiration) and congenital conditions such as cystic fibrosis and primary immunodeficiency, among others (Table 3). A chest x-ray may be considered in the work-up of a child with suspected asthma, particularly if the diagnosis is unclear or if the child is not responding as expected to treatment.

#### Diagnostic considerations in young children

The diagnosis of asthma in young children can be challenging since episodic respiratory symptoms, such as wheezing and cough, are also common in children without asthma [9]. In addition, spirometry is often unreliable in patients under 6 years of age, although it can be performed in some children as young as 5 years. A useful method of confirming the diagnosis in young children is a trial of treatment (8–12 weeks of a daily low-dose ICS and a short-acting bronchodilator as needed for rescue medication) [9, 10]. Improvement during the treatment period, as assessed by asthma control criteria (see Table 4), is an indicator that the daily ICS therapy is working and that a diagnosis of asthma is likely [9]. In a young child who is symptomatic with cough, wheeze, or increased difficulty breathing, a physical examination both before and after administration of a bronchodilator is of extreme value and can be used as a diagnostic tool. If the respiratory symptoms resolve within 10–15 min of bronchodilator administration, a diagnosis of asthma may be established.

**Table 4:** Criteria for assessing asthma control and risk factors for exacerbations [9, 10]

**Well-controlled asthma criteria**
· No exacerbations · Fewer than 2 doses per week of a rescue inhaler (SABA) · Daytime symptoms < 2 days per week · No nighttime symptoms or nocturnal awakenings due to asthma · Normal physical activity · No absenteeism from work or school · FEV_1_ or PEF at least 90% of personal best
**Risk factors for exacerbations**
Factors that increase the risk of exacerbations even if the patient has few asthma symptoms: · SABA over-use: High SABA use (≥ 3 × 200-dose canisters/year is associated with increased risk of exacerbations and increased mortality particularly if ≥ 1 canister per month) · Inadequate ICS: Not prescribed ICS, poor adherence, or incorrect inhaler technique · Other medical conditions: Obesity, chronic rhinosinusitis, GERD, confirmed food allergy, pregnancy · Exposures: Smoking, e-cigarettes, cannabis, allergen exposure if sensitized, air pollution · Psychosocial: Major psychological or socioeconomic problems · Lung function: Low FEV_1_ (especially < 60% predicted), high bronchodilator responsiveness · Type 2 inflammatory markers: Higher blood eosinophils, high FeNO (adults with allergic asthma on ICS) · Exacerbation history: Ever intubated or in intensive care unit for asthma; ≥ 1 severe exacerbation in last year

FeNO: fractional exhaled nitric oxide, FEV_1_: forced expiratory volume in 1 s, GERD: gastroesophageal reflux disease, ICS: inhaled corticosteroids PEF: peak expiratory flow, SABA: short-acting beta2 agonist

### Management

The goal of asthma management is to achieve the best possible asthma outcomes for each patient, including long-term symptom control (i.e., few/no asthma symptoms, no sleep disturbance, unimpaired physical activity) and long-term asthma risk minimization (i.e., no exacerbations, improved or stable personal best lung function, no requirement for maintenance OCS, no medication side effects) [9]. The core components of asthma management include: assessing asthma control and risk of exacerbation, providing asthma self-management education including a written action plan, identifying triggers and discussing environmental control if applicable, and medical management [9, 10].

#### Assessing asthma control and exacerbation risk

Criteria for asthma control are shown in Table 4 [9, 10]. According to current guidelines, assessing symptom control alone is not sufficient since patients with few asthma symptoms can still have severe or fatal exacerbations related to individual risk factors or external triggers (e.g., viruses, allergen, pollution) [9, 10]. Therefore, the patient’s risk factors for exacerbations (see Table 4), along with asthma control, should be assessed at each clinical encounter.

#### Asthma education and written action plan

Asthma patients must be empowered to take an active role in the management of their disease. Therefore, it is important that patients and families receive appropriate asthma self-management education as well as a personalized written action plan [9, 10]. Examples of asthma action plans can be found at https://asthma.ca/ (see Resources section) and https://lunghealth.ca (see Resource Library).

#### Identifying triggers and discussing environmental control

Environmental factors that trigger a patient’s asthma should be identified on history and avoided, if possible. Avoidance of exposure to irritants and relevant allergens is important for all patients with asthma. Irritants may include scented products (cosmetics, perfumes, cleaning products) or smoke (from fires, eCigarettes, tobacco, and marijuana). While irritants can act as triggers for asthma and rhinitis symptoms just as an allergen can, they are not IgE-mediated reactions. Skin prick testing is only helpful in identifying allergic triggers which are IgE-mediated.

#### Medical management

Medications used for the treatment of asthma can be classified as controllers (medications taken daily on a long-term basis that achieve control primarily through anti-inflammatory effects) and relievers (medications used on an as-needed basis for quick relief of bronchoconstriction and symptoms). Reliever medications include rapid-acting inhaled beta_2_-agonists and inhaled anticholinergics [9, 10]. Controller medications are summarized in Table 5 and include ICSs, LABAs in combination with an ICS, leukotriene receptor antagonists (LTRAs), long-acting muscarinic receptor antagonists (LAMAs), “triple therapy” with the combination of ICS/LABA/LAMA, and biologic agents [9, 10, 19, 21–26]. Allergen-specific immunotherapy may also be considered in most patients with identified environmental allergies but must be prescribed by physicians who are adequately trained in the treatment of allergies [9, 27] (see *Allergen Immunotherapy* article in this supplement) [28]. Systemic corticosteroid therapy may sometimes be required for the management of acute asthma exacerbations.

**Table 5:** Overview of the main controller therapies used for the treatment of asthma [19, 21–26]

	Adult dose information (≥ 18 years of age)	Pediatric dose information	
		< 6 years of age	6 – 17 years of age
**ICSs**			
Beclomethasone (Qvar, generics) pMDI	• *Low:* 50–100 µg bid • *Med:* 150–250 µg bid • *High:* > 250 µg bid (max 800 µg/day)	• *Low:* 50 µg bid • *Med:* 100 µg bid • *High:* refer to specialist *Approved age by Health Canada* ≥ *5 years*	• *Low:* 50–100 µg bid • *Med:* 150–250 µg bid • *High*: > 250 µg bid
Budesonide (Pulmicort) DPI	• *Low:* 100–200 µg bid • *Med:* 300–400 µg bid • *High:* > 400 µg bid (max 2400 µg/day)	DPI not recommended for children < 6 years	• *Low:* 100–200 µg bid • *Med:* 300–400 µg bid • *High:* > 400 µg bid *Approved age by Health Canada* ≥ *6 years*
Ciclesonide (Alvesco) pMDI	• *Low:* 100–200 µg once daily • *Med:* 400 µg once daily • *High:* > 400 µg once daily (max 800 µg/day)	Not recommended for children < 6 years	• *Low:* 100–200 µg once daily • *Med:* 400 µg once daily • *High:* > 400 µg once daily *Approved age by Health Canada* ≥ *6 years*
Fluticasone furoate (Arnuity Ellipta) DPI	• *Low:* 100 µg once daily • *High:* 200 µg once daily (max 200 µg daily)	Not approved by Health Canada for children < 12 years	• *Low:* 100 µg once daily • *High:* 200 µg once daily *Approved age by Health Canada* ≥ *12 years*
Fluticasone propionate (Flovent HFA [pMDI], Flovent Diskus [DPI], Aermony RespiClick [DPI])	• *Low:* 50–100 µg bid • *Med:* 100–250 µg bid • *High:* > 250 µg bid (max 2000 µg/day)	• *Low:* 50 µg bid • *Med:* 100–125 µg bid • *High:* refer to specialist *Approved age by Health Canada* ≥ *1 year for HFA (pMDI),* ≥ *4 years for Diskus (DPI)* *RespiClick not approved by Health Canada for children* < *12 years*	• *Low:* 50–100 µg bid • *Med:* 100–250 µg bid • *High:* ≥ 250 µg bid *RespiClick approved age by Health Canada for ages* ≥ *12 years*
Mometasone furoate (Asmanex) DPI	• *Low:* 100–200 µg daily • *Med:* 300–400 µg daily • *High:* > 400 µg µg daily (max 800 µg daily) Given once daily or bid	DPI not recommended for children < 6 years	• *Low:* 100–200 µg daily • *Med:* 300–400 µg daily • *High:* > 400 µg µg daily Given once daily or bid
**Combination ICS/LABA inhalers**			
Budesonide/formoterol (Symbicort) DPI	Maintenance: 100/6 µg or 200/6 µg, once daily or bid Maintenance and reliever: 200/6 µg as needed, max 8 inhalations/day	Not approved by Health Canada for children < 12 years	Maintenance: 100/6 µg or 200/6 µg, once daily or bid Maintenance and reliever: 200/6 µg as needed, max 8 inhalations/day *Approved age by Health Canada* ≥ *12 years*
Fluticasone propionate /salmeterol (Advair [pMDI], Advair Diskus [DPI])	pMDI: 2 puffs (250/50 – 500/50 µg) bid Diskus: 2 puffs (100/50—500/50 µg bid) (max 1000/100 µg/day)	Diskus (DPI): 1 puff (100/50 µg) bid *Approved age by Health Canada* ≥ *4 years for Diskus (DPI),* ≥ *12 years* *for pMDI*	pMDI: 2 puffs (250/50 – 500/50 µg) bid Diskus: 2 puffs (100/50—500/50 µg bid) (max 1000/100 µg/day) *Approved age by Health Canada* ≥ *12 years for pMDI*
Fluticasone furoate/vilanterol (Breo Ellipta) DPI	100/25 – 200/25 µg once daily (max 1 inhalation/day)	Not approved by Health Canada for < 18 years of age	
Mometasone/formoterol (Zenhale) pMDI	200/10—400/10 µg (2 puffs) bid Max 800/20 µg/day	Not approved by Health Canada for children < 12 years	200/10—400/10 µg (2 puffs) bid Max 800/20 µg/day Not approved by Health Canada for children < 12 years
Mometasone/indacaterol (Atectura Breezhaler) DPI	80/150 – 320/150 µg (1 inhalation) daily (max 320/150 µg/day)	Not approved by Health Canada for children < 12 years	80/150 – 320/150 µg (1 inhalation) daily (max 320/150 µg/day) Not approved by Health Canada for children < 12 years
**LTRAs**			
Montelukast (Singulair)	10 mg tablet od (taken in the evenings)	4 mg chewable tablet or granules od (taken in the evenings) *Approved age by Health Canada* ≥ *2 years*	5 mg chewable tablet od (6 – 14 years) 10 mg tablet od (≥ 15 years) (taken in the evenings)
**LAMAs**			
Tiotropium (Spiriva Respimat)	2 inhalation (5 µg) daily	Not approved by Health Canada for < 18 years of age	
**Combination ICS/LAMA/LABA inhalers**			
Fluticasone/umeclidinium/vilanterol (Trelegy) DPI	100/62.5/25—200/62.5/25 µg 1 inhalation daily	Not indicated for patients < 18 years	
Mometasone/glycopyrronium/indacaterol (Enerzair Breezhaler) DPI	160/50/150 µg 1 inhalation daily	Not indicated for patients < 18 years	
**Biologics**			
* Anti-IgE therapy*			
Omalizumab (Xolair)	150–375 mg sc every 2–4 weeks (based on patient’s weight and pre-treatment serum IgE level)	Not indicated for children < 6 years	75–375 mg sc every 2–4 weeks (based on patient’s weight and pre-treatment serum IgE level)
*Anti-IL5 therapy*			
Mepolizumab (Nucala)	100 mg sc every 4 weeks	Not indicated for asthma in children < 6 years	Children 6—11 years: 40 mg sc q4weeks Adolescents 12—17 years: 100 mg sc q4weeks
Reslizumab (Cinqair)	3 mg/kg IV every 4 weeks	Not indicated for children < 18 years	
Benralizumab (Fasenra)	30 mg sc every 4 weeks for the first 3 doses, then every 8 weeks thereafter	Not indicated for children < 18 years	
*Anti-IL4 / 13 therapy*			
Dupilumab (Dupixent)	600mg sc loading dose, then 300mg sc q2weeks OR 400mg sc loading dose, then 300mg sc q2weeks OR 400mg sc loading dose, then 200mg SC q2weeks	Not indicated for asthma in children < 6 years	Body weight > 60 kg: 200mg sc q2weeks Body weight 30—60 kg: 200mg sc q2weeks OR 300mg sc q4weeks Body weight 15—30 kg: 300mg sc q4weeks
*Anti-TSLP therapy*			
Tezepelumab (Tezspire)	210 mg sc q4weeks	Not indicated for asthma in children < 12 years	In children > 12 years: 210 mg sc q4weeks Not indicated for asthma in children < 12 years

*bid* twice daily, *DPI* dry powder inhaler, *ICS* inhaled corticosteroid, *IgE* immunoglobulin E, *IL* interleukin, *IV* intravenously, *LABA* long-acting beta agonist, *LAMA* long-acting muscarinic receptor antagonist, *LTRA* leukotriene receptor antagonists, *pMDI* pressurized metered-dose inhaler, *po* oral; *prn* as needed, *q* ever, *sc* subcutaneously, *TSLP* thymic stromal lymphopoietin

**Fig. 1:**
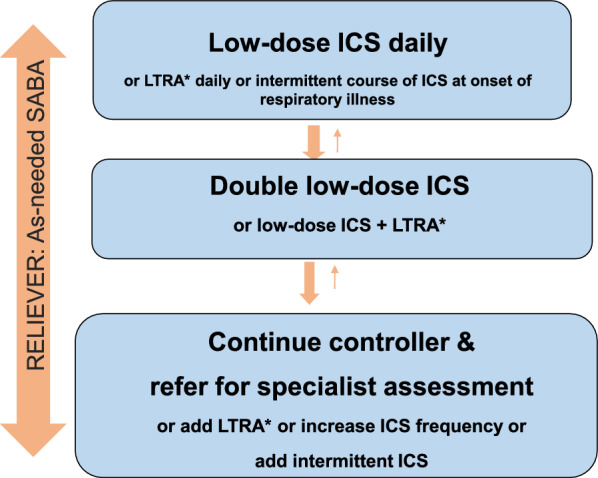
A simplified, stepwise algorithm for the treatment of asthma in children ≤ 5 years of age [9]**.** *If prescribing LTRA, advise about potential neuropsychiatric adverse effects. *ICS*: inhaled corticosteroid, *LTRA* leukotriene receptor antagonist, *SABA* short-acting beta_2_ agonist

**Fig. 2:**
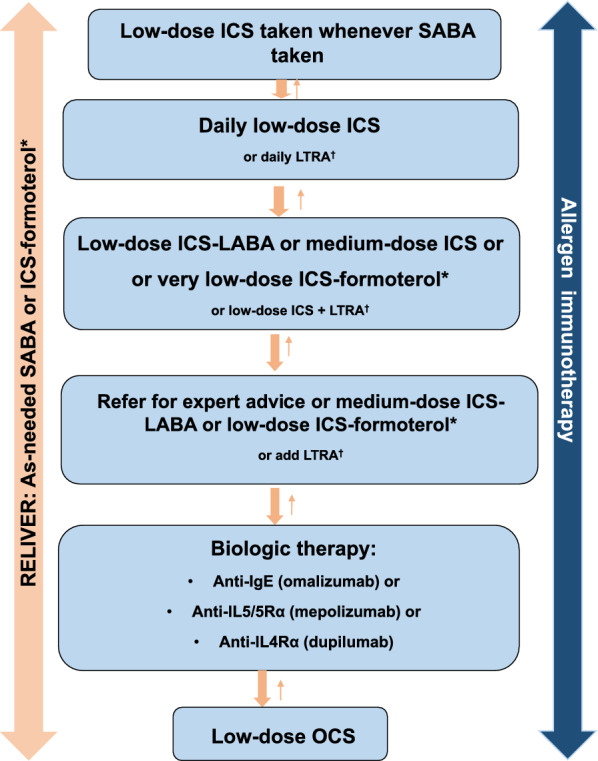
A simplified, stepwise algorithm for the treatment of asthma in children 6–11 years of age [9]. *In Canada, ICS-formoterol is not approved for children under 12 years of age although it is sometimes prescribed off-label for children 6–11 years of age ^†^If prescribing LTRA, advise about potential neuropsychiatric adverse effects. *ICS* inhaled corticosteroid, *IgE* immunoglobulin E, *IL* interleukin, *LABA* long-acting beta_2_ agonist, *LTRA* leukotriene receptor antagonist, *OCS* oral corticosteroid, *SABA* short-acting beta_2_ agonist

**Fig. 3:**
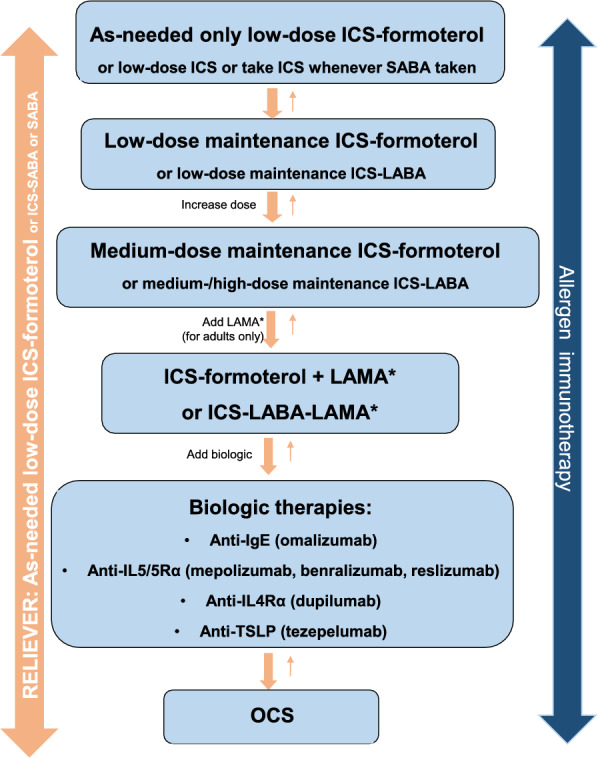
A simplified, stepwise algorithm for the treatment of asthma in adolescents and adults (≥ 12 years of age) [9]. *Indicated for patients ≥ 18 years of age. *ICS* inhaled corticosteroid, *LTRA* leukotriene receptor antagonist, *LABA* long-acting beta_2_-agonist, *IgE* immunoglobulin E, *IL* interleukin, *LAMA* long-acting muscarinic receptor antagonist, *SABA* short-acting beta2 agonist, *TSLP* thymic stromal lymphopoietin, *OCS* oral corticosteroids

Figures 1, 2 and 3 provide simplified, stepwise algorithms for the treatment of asthma in children (≤ 5 years of age and 6–11 years of age) and adolescents and adults (≥ 12 years of age). Patients should be treated in a step-wise fashion, with the fewest medications at the lowest dosage required to achieve control. Once asthma is well controlled, ongoing monitoring is essential. Asthma is a variable disease and treatment may need to be adjusted over time to maintain control [9, 10].

#### Inhaled medication delivery devices

Inhaled asthma medications come in a variety of forms including pressurized metered-dose inhalers (pMDIs) and dry powder inhalers (DPIs) (see Table 5). Not all medications are available in the same delivery devices. Also, some devices have dose counters included and others, such as pMDIs, do not. Shared-decision making should be employed in selecting an appropriate medication with consideration paid to technique, frequency of administration, and the need for single vs. multiple devices.

For patients ≥ 6 years of age, a pMDI plus spacer with mouthpiece or DPI is recommended [19]. Since children must have sufficient inspiratory force to use a DPI, these devices are generally not recommended for children under 6 years of age. In children, pMDIs must always be used with a spacer device. A spacer with face mask is recommended for children 2–4 years of age, while a spacer with mouthpiece is recommended for children 4–6 years of age. To transition to a spacer with mouthpiece, children must be able to form a seal around the mouthpiece and breathe through their mouth.

#### Reliever medications

All individuals with asthma should have access to a reliever for use as needed to treat acute symptoms [9, 10]. SABAs (e.g., salbutamol, terbutaline) or a combination inhaler (ICS-formoterol) are the preferred reliever medications for the treatment of acute symptoms. In Canada, as needed ICS-formoterol is approved for use as a reliever in adults and children ≥ 12 years of age, although it is sometimes used off-label in children ≥ 6 years of age. SABAs should only be taken on an as needed basis for symptom relief. Reliever medications do not need to be used routinely prior to the use of controller medications [10].

#### Controller medications

- *Inhaled corticosteroids (ICS) alone or in combination*

ICSs are the most effective anti-inflammatory medications available for the treatment of asthma and represent the mainstay of therapy [9, 10]. Regular ICS use has been shown to reduce symptoms and exacerbations, improve lung function and quality of life. There is no "cure" for asthma, and symptoms typically return within weeks to months after discontinuing ICS therapy. Since asthma is a chronic condition, most patients will require long-term, if not life-long, ICS treatment.

Regular daily low-dose ICS is recommended as first-line maintenance therapy for most children with asthma [9, 10] (see Figs. 1 , 2). For adolescents and adults, initiating treatment with a low-dose ICS in combination with a LABA is recommended [9, 10] (Fig. 1). In these patients, the ICS-formoterol combination is preferred as it has been shown to reduce the risk of severe exacerbations compared to regimens that use SABA as a reliever, with similar symptom control and lung function [9]. Furthermore, this treatment regimen is simpler, with patients using a single medication for reliever and for maintenance treatment.

Since ICSs are highly effective when used optimally, factors other than treatment efficacy need to be considered if ICS therapy is unsuccessful in achieving asthma control. These factors include: misdiagnosis of the disease, poor adherence to ICS therapy, improper inhaler technique, continued trigger exposure or the presence of other comorbidities. If, after addressing such factors, patients fail to achieve control with low-to-moderate ICS doses, then treatment should be modified. For most young children (≤ 5 years of age), ICS dose escalation (to a moderate dose) is generally the preferred approach to achieve control (see Fig. 1). For older children, adolescents and adults, the addition of another class of medication is generally recommended (see Figs. 2, 3). Young children who fail to achieve control on a moderate ICS dose, or older children, adolescents or adults with uncontrolled asthma despite moderate-to-high doses of ICS-formoterol or ICS/LABA therapy should be referred to an asthma specialist (i.e., respirologist or allergist).

The most common local adverse events associated with ICS therapy are oropharyngeal candidiasis (also known as oral thrush) and dysphonia (hoarseness, difficulty speaking). Rinsing and expectorating (spitting) after each treatment and the use of a spacer with pMDI devices can help reduce the risk of these side effects. Systemic adverse effects with ICS therapy are rare, but may occur at high doses, such as > 500 μg of fluticasone propionate equivalent, and include changes in bone density, cataracts, glaucoma and growth retardation [9]. Patients using high ICS doses should also be monitored for adrenal suppression [29]. The potential for side effects with ICS therapy needs to be considered in the context of other steroids (i.e., systemic, intranasal and topical) that may be prescribed for other atopic conditions such as allergic rhinitis or atopic dermatitis.

- *Leukotreine receptor antagonists (LTRA)*

The LTRA, montelukast, is also effective for the treatment of asthma and is generally considered to be safe and well tolerated. Because LTRA are less effective than ICS treatment when used as monotherapy, they are generally recommended as add-on therapy if asthma is uncontrolled despite the use of low-to-moderate dose ICS therapy in children or combination ICS/LABA or ICS-formoterol therapy in adolescents and adults. However, a black box warning has been issued for LTRAs due to neuropsychiatric side effects, most commonly irritability, aggressiveness, anxiety and sleep disturbance including suicidal thoughts or actions [10, 30]. This needs to be considered when prescribing LTRA in patients with asthma.

- *Long-acting muscarinic antagonists (LAMA)*

The LAMA, tiotropium, administered by mist inhaler can be used as add-on therapy for adult patients with a history of exacerbations despite treatment with ICS/LABA or ICS-formoterol combination therapy [9, 10]. LAMAs are only indicated for patients 18 years of age and older. In Canada, two ICS/LABA/LAMA “triple” therapies in a single inhaler are approved for adults 18 years of age and older (see Table 5).

- *Biologic therapies*

Biologics are potent immunomodulatory medications that treat severe asthma by precisely targeting excessive inflammation contributing to airway disease. There are currently 6 biologic therapies approved for the treatment of severe asthma in Canada that target one or more of the inflammatory mediators involved in the pathogenesis of asthma (see Tables 5 and 6). Omalizumab is an anti-IgE monoclonal antibody that is indicated for moderate to severe persistent asthma in patients with a positive skin test or in vitro reactivity to a perennial aeroallergen and whose symptoms are inadequately controlled with ICS therapy [21]. Dupilumab is a monoclonal antibody that targets the IL-4 receptor alpha subunit, inhibiting IL-4 and IL-13 signaling. It is indicated for severe asthma with a type 2/eosinophilic phenotype or OCS-dependent asthma [22]. The anti-IL-5 monoclonal antibodies, mepolizumab and reslizumab, and the monoclonal antibody targeting the IL-5 receptor, benralizumab, are indicated for the treatment of severe eosinophilic asthma [23–25]. Tezepelumab is a monoclonal antibody that targets thymic stromal lymphopoietin (TSLP), and it is indicated for severe asthma without phenotype or biomarker limitations [26]. Table 6 provides an overview of the Canadian indications for these biologic therapies.

**Table 6:** Biologics for severe asthma in Canada: indications [21–26]

	Omalizumab (Xolair)	Benralizumab (Fasenra)	Mepolizumab (Nucala)	Reslizumab (Cinqair)	Dupilumab (Dupixent)	Tezepelumab (Tezspire)
**Asthma indication**	Moderate-to-severe persistent asthma	Add-on maintenance treatment in severe eosinophilic asthma			Add-on maintenance in severe asthma with type 2/ eosinophilic phenotype or OCS-dependence	Add-on maintenance in severe asthma
**Age indication**	≥ 6 years	≥ 18 years	≥ 6 years	≥ 18 years	≥ 6 years	≥ 12 years
**Other requirements in indication**						
** Eosinophil count**	**―**	No cut-off	≥ 150 cells/µL at initiation or ≥ 300 cells/µL in past 12 months	≥ 400 cells/µL at initiation	No cut-off	No cut-off
**Control status**	Inadequately controlled with ICS	―	Inadequately controlled with high-dose ICS (≥ 18 years) or medium-to-high dose ICS (6–17 years) + additional controller (e.g., LABA)	Inadequately controlled with medium-to-high dose ICS + additional controller (e.g., LABA)	―	**―**
**Allergic component**	Positive skin test OR in vitro reactivity to a perennial aeroallergen	―	―	―	―	**―**
**Other indications:**						
	· CRSwNP · Chronic idiopathic urticaria	―	· CRSwNP · EGPA · HES	―	· Atopic dermatitis · CRSwNP · EoE · Prurigo nodularis	**―**

*CRSwNP* chronic rhinosinusitis with nasal polyps, *EGPA* eosinophilic granulomatosis with polyangiitis, *EoE* eosinophilic esophagitis, *HES* hypereosinophilic syndrome, *ICS* inhaled corticosteroids, *LABA* long-acting beta_2_-agonist, *OCS* oral corticosteroids

These biologics have revolutionized the management of severe asthma due to their efficacy, safety, and ability to reduce the need for OCS among patients with severe disease [31, 32]. Selecting a biologic involves consideration of the patient’s asthma phenotype and co-morbid atopic conditions [32] such as atopic dermatitis (see *Atopic Dermatitis* article in this supplement) [3], food allergy (see *IgE-mediated Food Allergy* paper in this supplement) [33], CRSwNP, chronic urticaria (see *Urticaria* article in this supplement) [34], and eosinophilic esophagitis (see *Eosinophilic Esophagitis* article in this supplement) [35]. Access to biologic therapy can be challenging due to the high cost of these medications, however they are now listed on many provincial formularies and should be considered in all patients with severe asthma who meet the inclusion criteria. An asthma expert, such as an allergist, can help determine which biologic is best for individual patients with severe asthma based on their phenotype.

#### Systemic corticosteroids

Systemic corticosteroids, such as oral prednisone, are generally used for the acute treatment of moderate to severe asthma exacerbations. While chronic systemic corticosteroid therapy may also be effective for the management of difficult to control asthma, prolonged use of oral steroids is associated with well-known and potentially serious adverse effects and, therefore, their routine or long-term use should be avoided if at all possible, particularly in children [9]. Adverse events with systemic corticosteroid use can occur with short- or long-term use although the risk is higher with long-term use. Adverse events can occur in multiple organ systems. Of most concern, is the increased risk of infection and cardiovascular events [36]. An observational study found an increased risk of death due to predominantly cardiovascular events in patients using systemic corticosteroids [37].

#### Allergen immunotherapy

Allergen immunotherapy involves the subcutaneous or sublingual administration of gradually increasing quantities of the patient’s relevant allergens until a dose is reached that is effective in inducing immunologic tolerance to the allergen. Subcutaneous immunotherapy has been shown to be effective against allergic asthma caused by grass, ragweed, HDM, cat and dog dander, and Alternaria [27, 38]. A Cochrane review of 88 randomized controlled trials examining the use of subcutaneous immunotherapy in asthma management confirmed its efficacy in reducing asthma symptoms and the use of asthma medications, and improving airway hyperresponsiveness [39]. Similar benefits on asthma outcomes have been noted with sublingual immunotherapy [40, 41], which is available for HDM, grass, birch and ragweed allergies (see *Allergen Immunotherapy* article in this supplement) [28]. Evidence also suggests that allergen immunotherapy may prevent the onset of asthma in atopic individuals [42, 43].

Allergen immunotherapy may be considered as add-on therapy for all patients 6 years of age or older with asthma who have clinically significant sensitization to aeroallergens [9]. However, it is contraindicated for patients with poorly controlled or severe asthma [27]. Since allergen immunotherapy carries the risk of anaphylactic reactions, it should only be prescribed by physicians who are adequately trained in the treatment of allergy and the use of immunotherapy (such as allergists and immunologists).

#### Indications for referral

In older children, adolescents and adults, referral to a specialist in asthma care (e.g., respirologist, allergist) is recommended when:

- Atypical asthma symptoms are present, or the diagnosis of asthma is in question;
- The patient has poor asthma control (poor lung function, persistent asthma symptoms) or severe asthma exacerbations (≥ 1 course of systemic steroids per year or hospitalization) despite moderate doses of ICS in children or moderate-to-high ICS/LABA or ICS-formoterol doses in adolescents and adults (with proper technique and good compliance);
- The patient requires a detailed assessment for and management of potential environmental triggers;
- The patient has been admitted to the intensive care unit (ICU) for asthma.

To find a local allergist/immunologist, clinicians can go to the Canadian Society of Allergy & Immunology (CSACI) Find an Allergist website at https://www.csaci.ca/find-an-allergist/.

## Conclusions

Asthma is one of the most common respiratory disorders in Canada and contributes to significant morbidity and mortality. A diagnosis of asthma should be suspected in patients with recurrent cough, wheeze, chest tightness and dyspnea, and should be confirmed using objective measures of lung function (spirometry preferred). Allergy testing is also recommended to identify possible triggers of asthma symptoms.

In most patients, asthma control can be achieved using avoidance measures and appropriate pharmacological interventions that include ICSs (either alone or in combination) as the mainstay of therapy. Combination ICS/LABA/LAMA inhalers or biologic therapies may be useful in select cases of difficult to control asthma. Allergen immunotherapy is a potentially disease-modifying therapy but should only be prescribed by physicians with appropriate training in allergy. All patients with asthma should have regular follow-up visits during which criteria for asthma control, adherence to therapy and proper inhaler technique should be reviewed.

## Data Availability

Not applicable.

## Abbreviations

AERD: Aspirin-exacerbated respiratory disease
bid: Twice daily
CHF: Congestive heart failure
COPD: Chronic obstructive pulmonary disease
CRSwNP: Chronic rhinosinusitis with nasal polyposis
CTS: Canadian Thoracic Society
DPI: Dry powder inhaler
FeNO: Fractional exhaled nitric oxide
FEV_1_: Forced expiratory volume in one second
FVC: Forced vital capacity
GERD: Gastroesophageal reflux disease
GINA: Global Initiative for Asthma
ICS: Inhaled corticosteroid
ICU: Intensive care unit
IgE: Immunoglobulin E
IL: Interleukin
IV: Intravenously
LABA: Long-acting beta_2_-agonist
LAM: Lymphangioleiomyomatosis
LTRA: Leukotriene receptor antagonist
LAMA: Long-acting muscarinic receptor antagonist
MAIT: Mucosal-associated invariant T
NERD: Non-steroidal anti-inflammatory drug-exacerbated disease
NK: Natural killer
OCS: Oral corticosteroids
od: Once daily
PC: Provocative concentration
PD: Provocative dose
PEF: Peak expiratory flow
pMDI: Pressurized metered dose inhalers
SABA: Short-acting beta_2_-agonist
sc: Subcutaneously
Th2: T helper cell type-2
TSLP: Thymic stromal lymphopoietin
